# Increase of Chamazulene and α-Bisabolol Contents of the Essential Oil of German Chamomile (*Matricaria chamomila* L.) Using Salicylic Acid Treatments under Normal and Heat Stress Conditions

**DOI:** 10.3390/foods5030056

**Published:** 2016-08-27

**Authors:** Mojtaba Ghasemi, Nadali Babaeian Jelodar, Mohammad Modarresi, Nadali Bagheri, Abbas Jamali

**Affiliations:** 1 Department of Research and Development, Sea Bioprospecting Co., Ltd., Tangestan Growth and Technology Center, Persian Gulf Science and Technology Park, Bushehr 7515797414, Bushehr, Iran; 2 Department of Plant Breeding and Biotechnology, Faculty of Crop Science, Sari Agricultural Sciences and Natural Resources University, Sari 578, Mazandaran, Iran; n.babaeian@sanru.ac.ir (N.B.J.); n.bagheri@sanru.ac.ir (N.B.); 3 Department of Plant Breeding, Faculty of Agriculture and Natural Resources, Persian Gulf University, Bushehr 7561158818, Bushehr, Iran; modarresi@pgu.ac.ir; 4 The Persian Gulf Research and Study Institute, Persian Gulf University, Bushehr 7561158818, Bushehr, Iran; a.jamali111@yahoo.com; 5 Young Researchers and Elite Club, Bushehr Branch, Islamic Azad University, Bushehr 751961955, Iran

**Keywords:** heat stress, salicylic acid, chamazulene, α-bisabolol, chamomile, essential oil, GC-MS

## Abstract

The chamazulene and α-(−)-bisabolol contents and quality of the chamomile oil are affected by genetic background and environmental conditions. Salicylic acid (SA), as a signaling molecule, plays a significant role in the plant physiological processes. The aim of this study was to evaluate the chemical profile, quantity, and improve the essential oil quality as a consequence of the increase of chamazulene and α-(−)-bisabol using salicylic acid under normal and heat stress conditions by the gas chromatography-mass spectrometry (GC-MS) technique. The factorial experiments were carried out during the 2011–2012 hot season using a randomized complete block design with three replications. The factors include four salicylic acid concentrations (0 (control), 10, 25 and 100 mg·L^−1^), and three chamomile cultivars (Bushehr, Bona, Bodegold) were sown on two different planting dates under field conditions. Fourteen compounds were identified from the extracted oil of the samples treated with salicylic acid under normal and heat stress conditions. The major identified oil compositions from chamomile cultivars treated with salicylic acid were chamazulene, α-(−)-bisabolol, bisabolone oxide, β-farnesene, en-yn-dicycloether, and bisabolol oxide A and B. Analysis of variance showed that the simple effects (environmental conditions, cultivar and salicylic acid) and their interaction were significant on all identified compounds, but the environmental conditions had no significant effect on bisabolol oxide A. The greatest amount of chamazulene obtained was 6.66% at the concentration of 10 mg·L^−1^ SA for the Bona cultivar under heat stress conditions, whereas the highest α-(−)-bisabolol amount attained was 3.41% at the concentration of 100 mg·L^−1^ SA for the Bona cultivar under normal conditions. The results demonstrated that the application of exogenous salicylic acid increases the quantity and essential oil quality as a consequence of the increase of chamazulene and α-(−)-bisabolol under normal and heat stress conditions.

## 1. Introduction

Chamomile is an annual plant, belonging to Asteraceae family, indigenous to Iran and grows as a wild plant in Europe [1]. Chamomile is naturally dispersed in South, Southwest, West, and Northwest Iran and its utilization has a long history in Iranian traditional medicine in the form of chamomile tea [2,3,4]. The chamomile essential oil is extensively served in food, cosmetics, and pharmaceutical industries [5]. It is a popular treatment for numerous ailments, including sleep disorders, anxiety, digestion/intestinal conditions, skin infections/inflammation (including eczema), wound healing, infantile colic, teething pains, and diaper rash [6]. Many medical properties of chamomile are ascribed to its essential oil. Over 120 constituents have been recognized in chamomile essential oil, where α-(−)-bisabolol, chamazulene, β-farnesene, bisabolol oxides A and B, and α-bisabolone oxide A are the most important ones [4,7]. Active principles of German chamomile are terpenoids: α-bisabolol, α-bisabolol oxide A and B, chamazulene, and sesquiterpenes; coumarins: umbelliferone; flavonoids: luteolin, apigenin, and quercetin; spiroethers: en-yn dicycloether and other components such as tannins, anthemic acid, choline, polysaccharides, and phytoestrogens [8]. Sashidhara et al. (2006) reported that the oil components in wild and bred chamomile populations include the chamazulene (5.0%–24.0%), α-(−)-bisabolol (24.0%–41.5%), bisabolone oxide (2.0%–7.0%), bisabolol oxide A (1.0%–36.2%), and bisabolol oxide B (3.6%–20.42%). Overall, the sesquiterpenes constituent was more than 70% in the total essential oil of chamomile [9,10]. Tirillini et al. (2006) identified seventy-seven components in chamomile that include 99% of the essential oil. These components consist of chamazulene (8.4%), bisabolol oxide A (11.2%), Farnesene (71.1%) and Spathulenol (11.3%), and the oxygenated sesquiterpenes have the most content (42%) [11]. The investigation in Estonia on the essential oil of chamomile *(M. recutita)* indicated that the main constituents of the essential oils were as follows: bisabolol oxide A (39.4%), bisabolone oxide A (13.9%), (Z)-en-yn-dicycloether (11.5%), bisabolol oxide B (9.9%), α-bisabolol (5.6%), and chamazulene (4.7%) [8]. Another study revealed that the main sesquiterpenes in the chamomile essential oil consist of: chamazulene (19.9%), α-bisabolol (20.9%), bisabolol-oxides A and B (21.6% and 1.2%, respectively), and β-farnesen (3.1%). In lower concentrations α- and β-caryophyllene, caryophyllene-oxide and spathulenol, and also some monoterpenes like β-phellandrene (0.8%), limonene (0.8%), β-ocymene (0.4%), and γ-terpinen (0.2%) were identified [8]. Galambosi and Repcok (1991) reported that the chamazulene content was variable between 11% and 21% in four chamomile varieties during 1985–1989 in Finland [12]. Letchamo expressed that the bisabolol content increases until full blooming stage which is due to reduction in dicycloether content and has no relationship with metabolism of the other substances [13]. Mann and Staba obtained 0.4%–1.2% essential oil in chamomile flowers with bisabolol chemotype that contained 15%–30% α-(−)-bisabolol, but the real α-(−)-bisabolol content of essential oil depends on plant growth conditions [14]. The form, structure, and morphological traits of chamomile plants, their essential oil content, and quality are influenced by genetic makeup and environmental conditions [15]. D’Andrea reported that there were no statistical differences on the essential oil percentage among the four chamomile cultivars grown in southern Italy [16]. Variations in oil content and composition have been reported in essential oil–bearing plants such as basil and *Artemisia* under water stress conditions [7,17]. Rowshan and Bahmanzadegan reported that the the application of exogenous salicylic acid with 200 and 400 mg·L^−1^ concentrations may modify secondary metabolites and their pathway by impacts on plastids, the chlorophyll level, and representing stress conditions. The stress produced by SA modifies the quality and quantity of the essential oil of yarrow *(Achillea millefolium)* [18]. The content and compounds of essential oil are different in the chamomile flower and they depend on genotype, and environmental factors such as light intensity, day length, temperature, habitat, management of production and post-harvest processes [19,20]. Jeshni et al. indicated that drought stress caused significant effects on physiological traits, essential oil yield, and essential oil components. The essential oil components increased, whereas the essential oil yield decreased in response to severe drought stress [1]. In chamomile, the effects of cropping techniques, planting date, genotypes and ecological conditions on the yield of essential oil and the oil composition have been considered [7,20]. Farhoudi et al. indicated that medium drought stress increased the oil yield [2]. Ghasemi et al. reported that the exogenous application of salicylic acid concentrations improves the essential oil content of chamomile *(Matricaria chamomila* L.) under normal and heat stress conditions [20]. Soluble phenolic compounds increase in chamomile plants using salicylic acid (SA) [21]. Sadeghian et al. showed that foliar spray of SA at low concentration might be employed for enhancing both primary and secondary metabolites production of *S. khuzistanica* plants [22]. Rowshan et al. indicated that SA application manipulated essential oil components of *Salvia macrosiphon* [23]. Salicylic acid decreases negative effect of oxidative stress and it improves NaCl stress tolerance parameters accompany mineral nutrient contents in chamomile plants [24]. Drought stress decreases the agro-physiological parameters and apigenin content in German chamomile, but it has no significant affect on the essential oil [7]. The heat stress has been known as an agricultural issue in many arid and semi-arid areas all over the world. High temperature creates a series of physiological and biochemical modifications in plants, which affect plant growth and development and can lead to acute reduction in economic yield [20,25]. To investigate the effect of high temperature (heat stress) on growth and yield of plants under field conditions, several different warm locations, different sowing dates and/or controllable growth chambers are used [26,27,28,29]. Therefore, chamomile may be considered as an economical crop for environments with high temperature and water scarcity due to its considerable adaptability to a large spectrum of soils and climate conditions [7,30]. The objective of this research was to investigate the chemical profile, quantity, and improve the essential oil quality as a consequence of the increase of chamazulene and α-(−)-bisabolol using salicylic acid under normal and heat stress conditions, using the GC-MS technique.

## 2. Material and Methods

### 2.1. Field Experiments Description

Two factorial field experiments were performed using a randomized complete block design (RCBD) with three replications during the 2011–2012 hot season at the experimental field of the Bushehr Research Center for Agriculture and Natural Resources, Borazjan, Bushehr Province, Iran. The geographical coordinates of the experimental site was 29°12′21″ N, 51°15′07″ E, with an altitude of 110 m. The chamomile seeds were provided by the seed bank of the Medicinal Plants and Drugs Research Institute, Shahid Beheshti University, Tehran, Iran. The chemical and physical properties of the soil of the experimental location were presented in [20]. Each experimental plot size was 1 m × 1 m and in each plot, the plants were grown in three equidistant rows with adjacent rows being 30 cm apart. According to soil analysis, 20 g of ammonium nitrate fertilizer was used in each plot before the planting date, in addition to another 20 g being applied one month hence. Each experimental site had 36 plots (including 12 plots in each block) and, in total, the two experiments had 72 plots. The distance between the two main plots or experimental sites was seven meters. The seeds were sown directly and superficially by hand and then were covered through a very thin layer of sandy soil. The 15 plants were kept after seed germination and seedlings growing in each plot.

### 2.2. Experimental Design

The two field experiments consisted of three factors: (i) sowing dates (normal and stress); (ii) chamomile cultivars (Bushehr, Bona, and Bodegold); and (iii) salicylic acid concentrations (0, 10, 25, and 100 mg·L^−1^) were designed and performed. The above-mentioned factors were combined and the experiments were set up as a factorial scheme with 24 treatments (four salicylic acid levels × three cultivars × two sowing dates) replicated thrice in a randomized complete block design (RCBD).

### 2.3. Planting Dates and Air Temperature

In this research, the heat stress treatment was conducted under field conditions in a very hot area (Borazjan, Bushehr Province) in Southwest Iran by changing sowing dates (late planting date). The chamomile seeds were planted on two different sowing dates corresponding to an optimum planting date (24 December 2011) and late planting date (7 February 2012). The late planting date (heat stress induced by delayed sowing time) was set up so that more vegetative stages and complete blooming period were faced with high temperature at the end of agronomic season in Bushehr Province, Iran. The climatic conditions during the experimental year 2011–2012 were presented in [20].

### 2.4. Irrigation, and Weed and Pest Management

The irrigation was performed immediately after seed sowing using an installed pipeline and dropping-tube system. The normal and heat stress sites were irrigated every four days during cool months (normal site: January, February and early-March 2012; heat stress site: February until early-March 2012) in the morning at 10:00 a.m. Thereafter, with the beginning the warm season, both sites were irrigated in the morning and evening every day at 9:00 a.m. until 12:00 p.m. and 15:00 p.m. until 18:00 p.m., simultaneously. All plots were irrigated completely and uniformly. The chemical properties of used water in the experiment were presented in [20]. The weed management was done three times for normal and heat stress conditions during vegetative and reproductive phases by hand. Herbicide and pesticide were not used during this experiment and also the plant disease was not observed in the field.

### 2.5. Salicylic Acid Treatments

Salicylic acid treatments including four levels (0 (control), 10, 25, and 100 mg·L^−1^) were applied on three chamomile cultivars (Bushehr (diploid) with Iranian origin; Bona (diploid) with Slovakian origin; and Bodegold (tetraploid) with German origin). Salicylic acid was purchased from Merck Co. (Darmstadt, Germany). Foliar spray using salicylic acid was scheduled during the growing and flowering stages of chamomile plants. The salicylic acid treatments were applied three times during vegetative and reproductive phases at every 15 days in accordance with [20]. The first stage of salicylic acid foliar spraying was at 60 days after planting. The SA spraying was performed at 10:00 a.m. until 14:00 p.m. for each treatment and plot. All spraying solutions were applied to the shoots uniformly using a hand pump sprayer. The volume of salicylic acid sprayed was 1.1 L for each plot.

### 2.6. Harvest Management and Essential Oil Extraction

The flower harvest was carried out by hand and each time just the flowers were picked. The harvest times of chamomile cultivars under normal and heat stress conditions were presented in [20]. The chamomile flowers were dried at room temperature (20–25 °C) after each harvest time. The harvest was carried out every 7–10 days for each plot. All harvested samples (harvested times) were mixed together for each treatment and replication carefully, and then samples were powdered by an electrical mill machine in order to prepare for essential oil extraction. In order to extract chamomile oil, 30 g of air-dried flowers powder was weighed carefully by a precise digital balance device (±0.001 g) and then 300 mL distilled water was added to dried flowers powder with a 1:10 ratio in a 500-mL round-bottom flask. Thereafter, the essential oil of air-dried flowers of chamomile was isolated by hydro-distillation for 5 h, using a Clevenger-type apparatus according to the method described in British Pharmacopeia [7]. The essential oil was stored in dark glass bottle and then was dried over anhydrous sodium sulfate (Na_2_SO_4_). Finally, the essential oils were kept in refrigerator (4 °C) until they were analyzed [31].

### 2.7. GC-MS Analysis Conditions

Analysis and identification of the phytochemical compositions of the chamomile oils were conducted by using gas chromatography-mass spectrometry (GC-MS). The analysis was performed using an Agilent 7890 A gas chromatograph (Agilent Technologies, Palo Alto, CA, USA) coupled with an Agilent 5975 C mass spectrometry (Agilent Technologies, Palo Alto, CA, USA) equipped with a fused silica capillary HP-5 column (30 m length × 0.25 mm i.d., 0.25 μm film thickness). Helium was used as the carrier gas at a flow rate of 1.1 mL·min^−1^. The oven temperature program started at 70 °C and was held for 1 min. Then the column was sequentially heated at a rate of 10 °C·min^−1^ to 155 °C and was held for 0 min. Thereafter, the column was heated at a rate of 4 °C·min^−1^ to 210 °C and it was held for 1 min. Eventually, the column was heated at a rate of 8 °C·min^−1^ to 270 °C and was held for 2 min. The split ratio was 1:50 with ionization voltage of 70 eV. Both the transfer line temperature and the injector temperature were programmed at 280 °C and 250 °C, respectively.

### 2.8. Identification and Quantification of the Oil Compositions

Quantitative analyses of the main compounds such as chamazulene, trans-β-farnesene, α-bisabolol oxide (A, B), bisabolone oxide, en-yn-dicycloether, and spathulenol were performed using an internal standard (n-hexadecane). The dilution of the oil samples was performed by addition of 3 mL of n-hexane solvent to each sample and then 1 μL of each sample was injected to GC-MS apparatus. The components of the essential oils were identified by calculation of their retention indices (RI) relative to n-alkanes (C_10_–C_24_) with those of authentic compounds available in the laboratory. Further identification was made by matching the mass spectral fragmentation patterns of different compounds with corresponding data (Adams and Wiley 7.0 library) and other published mass spectra [32]. The relative percentage of the oil constituents was calculated from the GC peak area [5]. Finally, the 72 essential oil samples were injected and analyzed for each treatment in both environmental conditions (normal and heat stress). The essential oil samples were injected to the GC-MS apparatus one time.

### 2.9. Statistical analysis

After identification and measurement of the chemical compounds, all data were subjected to statistical analysis (ANOVA) using MSTAT-C and DSAASTAT software version 1.022 (Perugia, Italy) [33]. Means comparisons were performed by Duncan’s multiple range test at 5% level.

## 3. Results

**Figure 1:**
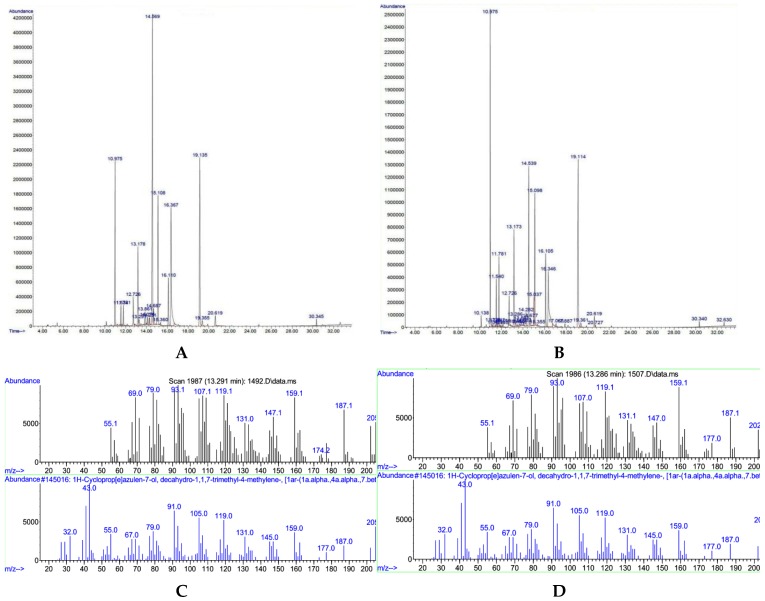
(**A**) A typical GC chromatogram for the Bodegold cultivar treated with 25 mg·L^−1^ SA under normal conditions; (**B**) a typical GC chromatogram for the Bodegold cultivar treated with 25 mg·L^−1^ SA under heat stress conditions; (**C**) a typical MS spectrum for chamazulene compound in the Bodegold cultivar treated with 25 mg·L^−1^ SA under normal conditions; and (**D**) a typical MS spectrum for chamazulene compound in the Bodegold cultivar treated with 25 mg·L^−1^ SA under heat stress conditions.

The hydro-distillation of the air-dried chamomile flowers gave dark blue oils in range of 0.1% to 0.8% (*w/w*) in this experiment. The compositions of the attained essential oils of chamomile are presented in Table 1. In total, fourteen components were identified in three cultivar of chamomile treated with salicylic acid under normal and stress conditions. According to the GC-MS results, the amount of oil components such as chamazulene was changed using salicylic acid treatments under normal and heat stress conditions (Figure 1).

The analysis of variance showed that all the presented compounds at Section 3.1, Section 3.2, Section 3.3, Section 3.4, Section 3.5, Section 3.6, Section 3.7, Section 3.8, Section 3.9, Section 3.10, Section 3.11, Section 3.12, Section 3.13 and Section 3.14 were significantly influenced by environmental conditions, chamomile cultivars, and salicylic acid treatments (Table 2), and the interaction of environmental conditions × cultivar, environmental conditions × salicylic acid, cultivar × salicylic acid, and the triple interaction of environmental conditions × cultivar × salicylic acid had a significant effect on them at the statistical level (*p* ≤ 0.01) (Table 2). Furthermore, the simple effects of environmental conditions, chamomile cultivars, salicylic acid treatments, and their interactions are presented in Tables S1 and S2.

### 3.1. Trans-*β*-Farnesene

The mean comparison for the triple interaction of environmental conditions × cultivar × salicylic acid indicated that the Bona cultivar had the highest amount of *trans*-β-farnesene percent, with an average of 19.68% at the concentration of 10 mg·L^−1^ SA under normal conditions, whereas the Bona cultivar had the lowest amount of *trans*-β-farnesene percent, with an average of 2.05% at the concentration of 25 mg·L^−1^ SA under heat stress conditions (Table 3).

### 3.2. Germacerene D

The mean separation for the triple interaction of environmental conditions × cultivar × salicylic acid showed that the Bona cultivar had the highest amount of germacerene D, with an average of 3.01% at the concentration of 10 mg·L^−1^ SA under heat stress conditions, whereas the Bona cultivar had the lowest amount of germacerene D, with an average of 0.04% at the concentration of 25 mg·L^−1^ SA under heat stress conditions (Table 3).

### 3.3. Germacerene B

The Duncan analysis for the triple interaction of environmental conditions × cultivar × salicylic acid demonstrated that the Bona cultivar had the highest amount of germacerene B, with an average of 4.95% at the concentration of 10 mg·L^−1^ SA under normal conditions, whereas the Bona cultivar had the lowest amount of germacerene B, with an average of 0.06% at the concentration of 25 mg·L^−1^ SA under heat stress conditions (Table 3).

### 3.4. Nerolidol

The mean results illustrated that the Bodegold cultivar had the highest amount of nerolidol percent, with an average of 3.76% at the concentration of 100 mg·L^−1^ SA under heat stress conditions, whereas the Bona cultivar had the lowest amount of nerolidol percent, with an average of 0.11% at the concentration of 100 mg·L^−1^ SA under normal conditions (Table 3).

### 3.5. Spathulenol

According to the Table 3, the Bodegold cultivar had the highest amount of spathulenol, with an average of 9.46% at the concentration of 100 mg·L^−1^ SA under normal conditions, whereas the Bodegold cultivar had the lowest amount of spathulenol percent, with an average of 0.00% at the concentration of 10 mg·L^−1^ SA under normal and heat stress conditions.

### 3.6. Bisabolol Oxide B

Regarding to the Table 3, the highest amount of bisabolol oxide B, with an average of 35.70% at the concentration of 0 mg·L^−1^ SA is achieved for Bodegold cultivar under normal conditions, whereas the lowest amount is achieved for Bona cultivar, with an average of 3.18% at the concentration of 25 mg·L^−1^ SA under heat stress conditions (Table 3).

### 3.7. 3-Methyl-Thiophene-2-Carboxamide A

As can be seen from Table 3, the Bushehr cultivar had the highest amount of 3-Methyl-thiophene-2-carboxamide A, with an average of 2.45% at the concentration of 10 mg·L^−1^ SA under heat stress conditions, whereas the Bona cultivar had the lowest amount, with an average of 0.65% at the concentration of 25 mg·L^−1^ SA under normal conditions.

### 3.8. α-Bisabolol

The mean separation for the triple interaction of environmental conditions × cultivar × salicylic acid α-bisabolol percent demonstrated that the Bona cultivar had the highest amount of α-bisabolol percent, with an average of 3.41% at the concentration of 100 mg·L^−1^ SA under normal conditions, whereas the Bushehr cultivar had the lowest amount of α-bisabolol percent, with an average of 0.02% at the concentration of 25 mg·L^−1^ SA under heat stress conditions (Table 3).

### 3.9. Bisabolone Oxide

The mean comparison of the triple interaction of environmental conditions × cultivar × salicylic acid on bisabolone oxide percent showed that the Bona cultivar had the highest amount of bisabolone oxide percent, with an average of 39.97% at the concentration of 25 mg·L^−1^ SA under normal conditions, whereas the Bodegold cultivar had the lowest amount, with an average of 8.35% at the concentration of 100 mg·L^−1^ SA under heat stress conditions (Table 3).

### 3.10. 3-Methyl-Thiophene-2-Carboxamide B

According to obtained means results from Table 3, the Bushehr cultivar had the highest amount of 3-Methyl-thiophene-2-carboxamide B percent, with an average of 0.62% at the concentration of 100 mg·L^−1^ SA under heat stress conditions, whereas the Bona and Bodegold cultivars had the lowest amount, with an average of 0.00% at the concentrations of 10 and 100 mg·L^−1^ SA under normal conditions, respectively.

### 3.11. Chamazulene

The means separation test showed that the triple interaction of environmental conditions × cultivar × salicylic acid on the Bona cultivar had the highest amount of chamazulene, with an average of 6.66% at the concentration of 10 mg·L^−1^ SA under heat stress conditions, whereas the Bodegold cultivar had the lowest amount, with an average of 1.05% at the concentration of 25 mg·L^−1^ SA under heat stress conditions (Table 3). A typical GC chromatogram of German chamomile oil and MS spectrum of chamazulene are presented for the Bodegold cultivar treated with 25 mg·L^−1^ SA under normal and heat stress conditions (Figure 1).

### 3.12. Bisabolol Oxide A

The means comparisons for the triple interaction of environmental conditions × cultivar × salicylic acid indicated that the Bodegold cultivar had the highest amount of bisabolol oxide A, with an average of 51.31% at the concentration of 10 mg·L^−1^ SA under heat stress conditions, whereas the Bodegold cultivar had the lowest amount, with an average of 7.31% at the concentration of 100 mg·L^−1^ SA under normal conditions (Table 3).

### 3.13. Cis-En-Yn-Dicycloether

The means results showed that the Bodegold cultivar had the highest amount of *cis*-en-yn-dicycloether percent with an average of 24.25% at the concentration of 100 mg·L^−1^ SA under heat stress conditions, whereas the Bushehr cultivar had the lowest amount with an average of 9.72% at the concentration of 0 mg·L^−1^ SA under normal conditions (Table 3).

### 3.14. Trans-En-Yn-Dicycloether

According to Duncan analysis, the highest amount of *trans*-en-yn-dicycloether, with an average of 1.42% at the concentration of 0 mg·L^−1^ SA is obtained for the Bona cultivar under normal conditions, whereas the lowest amount is obtained for the Bodegold cultivar, with an average of 0.33% at the concentration of 100 mg·L^−1^ SA under normal conditions (Table 3).

## 4. Discussion

The extracted essential oil had a blue to dark blue color in the present study [25,34]. The blue color of the essential oil is ascribed to the presence of chamazulene [35]. In total, fourteen costituents were identified in three cultivar of chamomile treated with salicylic acid under normal and stress conditions where chamazulene, α-(−)-bisabolol, bisabolol oxides A and B, farnesene, and α-bisabolone oxide A are the most important ones. Some factors, such as isolation method, environmental conditions (nutrient level, temperature), and some stresses may play a substantial role in the components and quality of extracted essential oil [23]. The delay in planting caused more oxygenated compounds to be produced due to high temperature and relatively long days. Flowering and the kind of essential oil profile are also genetically controlled, but their quantity depends on external factors. The response of chamomile cultivars were different to salicylic acid treatments under normal and heat stress conditions. The different response of chamomile cultivars is attributed to genotype and environmental conditions [19]. So that, the chamazulene and α-(−)-bisabolol contents were increased considerably in the Bona cultivar under heat stress and normal conditions, respectively. The application of exogenous salicylic acid at the specific concentration under heat stress and normal conditions was made increasing in the chamazulene and α-(−)-bisabolol contents, respectively. The production of medicinal plants is mostly dependant on ecological conditions. In this regard, management and monitoring of environmental parameters are very important [19]. Several studies indicated that environmental conditions had no, or only slight, influence on essential oil yield, as well as on chamazulene content, while the reaction of the bis-aboloids to these conditions was much more intense. However, they did not find any qualitative changes in essential oil composition due to experimental conditions [15,19]. The bisabolol content is related to growth environmental conditions, but chamazulene amount is more controlled genetically in the chamomile essential oil [14,19,36]. Exogenous application of salicylic acid enhanced plant tolerance to heat stress [20]. Additionally, it was effective in inducing secondary metabolites formation in plant cell culture or in vivo plants [23]. The chamazulene content is variable in different chamomile varieties during different years and climates [12]. The bisabolol content increases until the full blooming stage, which is due to reduction in dicycloether content and has no relationship with metabolism of the other substances [13]. More production of oxygenated compounds, i.e., α-bisabolol oxide A, α-bisabolol oxide B, and α-bisabolone oxide may be attributed to the effect of high temperatures by a delay in planting compared to earlier planting dates [19]. High temperatures at flowering by later planting dates in our experiment produced more trans-β-farnesene content compared to normal conditions which is in agreement to results of Rafieiolhossaini et al. (2010) [19]. Probably, the highest α-(−)-bisabolol content accumulates in chamomile flowers during sunshine and sunset time [14], which represent the effect of temperature conditions. In addition to the major influence of genetic factors, the environment has an important effect on essential oil accumulation and composition. The environmental control which is modified by chamomile plant ontogeny, and many other factors, such as light intensity, day length, temperature, nutrition, irrigation, plant growth regulators, tissue cultures and their transgenic transformation, intraspecific interactions, population dynamics, parasites, diseases, pest control, interspecific competition, and harvest management are also known to affect the yield of chamomile essential oil and its composition [37]. Results indicated the relative influence of environmental conditions and salicylic acid on essential oil, chamazulene, and α-bisabolol content. In general, the salicylic acid treatments modified the quantity and quality of the essential oil of chamomile and its constituents under normal and heat stress conditions.

## 5. Conclusions

Using the GC-MS technique, more reliable qualitative and quantitative analysis of complex essential oils samples could be carried out. The GC-MS analysis identified fourteen compounds in three cultivars of chamomile treated with salicylic acid under normal and stress conditions. The results indicated that the environmental conditions, cultivar, and salicylic acid effects, and their interaction, were significant on all identified compounds, but the environmental conditions had no significant effect on bisabolol oxide A. The greatest amount of chamazulene obtained was 6.66% at the concentration of 10 mg·L^−1^ SA for the Bona cultivar under heat stress conditions, while, the highest α-(−)-bisabolol amount attained was 3.41% at the concentration of 100 mg·L^−1^ SA for the Bona cultivar under normal conditions. In this experiment, Bona was the best cultivar under normal and heat stress conditions. It produced the highest amount of chamazulene and α-(−)-bisabolol using salicylic acid treatments. The content of chamazulene and α-bisabolol was increased using specific salicylic acid concentrations under normal and heat stress conditions. This increase may be related to the different response of the genotype to salicylic acid concentration and changes of the secondary metabolites pathway under different environmental conditions. The practical conclusion to be drawn from the experiment is that, on the basis of the data, it should be possible to produce chamomile flowers of more than adequate quality in the Bushehr region, as a very hot and arid zone in Southwest Iran. This investigation provides new knowledge for planting of German chamomile in a hot and arid zone, like South Iran. However, as far-reaching conclusions cannot be drawn just from the data of a single year, it should be emphasized that the results of this study have to be further confirmed. In order to produce the best yield with a good active principles profile, it is necessary to integrate a good genotype with optimal environmental (ecological) conditions. As oil composition between cultivars is very variable, it is important that the growing conditions be optimized for each particular cultivar. Selection for cultivation should be done according to the target compound and therapeutic value of chamomile. Therefore, the application of salicylic acid can be applied as a highly effective, economic, easy, and novel approach for improving the quantity and essential oil quality as a consequence of the increase of chamazulene and α-(−)-bisabol under normal and heat stress conditions. In addition, it sounds that more research is needed to explain the salicylic acid effect on the biochemical mechanisms of the essential oil components under normal and heat stress conditions.

## Figures and Tables

**Table 1:** Identified chemical compositions in the chamomile essential oil treated with salicylic acid under normal and heat stress conditions.

No.	Compounds	Retention Time (min)	Relative Retention Index
1	*Trans*-β-farnesene	10.97	1458
2	Germacerene D	11.53	1492
3	Germacerene B	11.78	1517
4	Nerolidol	12.72	1611
5	Spathulenol	13.17	1592
6	Bisabolol oxide B	14.54	1665
7	3-Methyl-thiophene-2-carboxamide A	14.68	1739
8	α-bisabolol	15.04	1685
9	Bisabolone oxide	15.15	1697
10	3-Methyl-thiophene-2-carboxamide B	19.37	1903
11	Chamazulene	16.12	1793
12	Bisabolol oxide A	16.42	1824
13	*Cis*-en-yn-dicycloether	19.12	1878
14	*Trans*-en-yn-dicycloether	19.37	1903

**Table 2:** Analysis of variance for identified chemical compounds of German chamomile cultivars treated with salicylic acid under normal and heat stress conditions.

Mean Square (MS)									
**S.O.V**	**DF**	***Trans*-β-farnesene (%)**	**Germacerene D (%)**	**Germacerene B (%)**	**Chamazulene (%)**	**α-Bisabolol (%)**	**Bisabolol Oxide B (%)**	**Bisabolone oxide (%)**	**Bisabolol oxide A (%)**
EC	1	383.31 **	8.36 **	27.48 **	2.57 **	0.27 **	564.74 **	265.74 **	0.92 ns
Cult	2	477.85 **	19.71 **	47.06 **	96.10 **	3.51 **	2305.12 **	2915.70 **	8401.82 **
SA	3	5.56 **	0.14 **	0.22 **	0.22 **	2.16 **	33.45 **	8.99 **	12.86 *
EC × Cult	2	205.62 **	6.96 **	23.44 **	4.69 **	2.40 **	225.75 **	348.10 *	584.83 **
EC × SA	3	10.12 **	0.08 **	0.22 **	1.03 **	2.75 **	11.76 **	13.97 **	26.01 **
Cult × SA	6	12.42 **	0.24 **	0.41 **	0.42 **	1.20 **	31.76 **	3.32 *	27.93 **
EC × Cult × SA	6	3.51 **	0.04 **	0.19 **	0.54 **	2.27 **	5.77 **	21.08 **	38.66 **
Error	48	0.16	0.0034	0.0087	0.031	0.0022	0.54	1.2	3.87
C.V. (%)		6.46	8.36	8.94	5.7	9.61	6.37	5.6	5.47
**S.O.V**	**DF**	**Nerolidol (%)**	**Spathulenol (%)**	**3-Methyl-thiophene-2-carboxamide A (%)**		***Cis*-en-yn-dicycloether (%)**	**3-Methyl-thiophene-2-carboxamide B (%)**		***Trans*-en-yn-dicycloether (%)**
EC	1	0.504 **	8.63 **	2.72 **		100.47 **	0.11 **		0.42 **
Cult	2	55.93 **	237.86 **	4.09 **		382.81 **	0.11 *		1.05 **
SA	3	0.15 **	1.06 **	0.08 **		18.60 **	0.09 **		0.19 **
EC × Cult	2	0.56 **	10.92 **	5.13 **		47.55 **	0.29 **		1.04 **
EC × SA	3	0.15 **	1.83 **	0.03 **		11.13 **	0.06 **		0.053 **
Cult × SA	6	00.24 **	2.36 **	0.05 **		7.81 **	0.02 **		0.049 **
EC × Cult × SA	6	0.16 **	0.96 **	0.15 **		8.92 **	0.03 **		0.13 **
Error	48	0.0067	0.041	0.0059		0.597	0.0002		0.0015
C.V. (%)		7.89	6.72	5.37		5.17	5.83		5.4

EC (environmental conditions); Cult (cultivar); SA (salicylic acid); symbol (*) and (**): indicates statistically significant differences between treatments at (*p* ≤ 0.05) and (*p* ≤ 0.01) levels, respectively, as well as the letters (ns) shows statistically non-significant differences between treatments.

**Table 3:** Mean comparison of the triple interaction of environmental conditions × cultivar × salicylic acid on chemical compounds of German chamomile.

Treatments		Chemical compounds													
		T-β-farn (%)	Germ-D (%)	Germ-B (%)	Chama (%)	α-Bisabolol (%)	Bisabolol oxide B (%)	Bisabolone oxide (%)	Bisabolol oxide A (%)	Nero (%)	Spath (%)	3-Methyl-A (%)	*Cis*-dicyclo (%)	3-Methyl-B (%)	*Trans*-dicyclo (%)
EC × Cult × SA	E1C1S1	2.13n	0.04j	0.09l	2.33ghi	0.10f	3.36kl	35.60b	44.13fgh	0.17ef	0.46k	1.07k	9.72l	0.45c	0.45jk
	E1C1S2	3.49jk	0.20hi	0.23jkl	2.31ghi	0.07f	12.25h	16.91fg	48.11cde	0.12f	2.72h	2.18bc	10.18kl	0.50b	0.80de
	E1C1S3	3.97j	0.56f	0.65ef	5.51c	1.48c	24.87c	11.90hij	22.29k	2.72c	4.67g	1.95e	18.32cde	0.36e	0.74ef
	E1C1S4	2.82klmn	0.09j	0.12kl	2.36gh	0.10f	3.13l	26.54d	50.04cd	0.12ef	0.45k	1.50hi	11.72ij	0.41d	0.69fg
	E1C2S1	7.11f	0.38g	0.47gh	1.52mn	0.10f	8.86i	16.56fg	42.25ghi	0.13ef	2.35ij	1.63gh	17.11e	0.21hij	1.42a
	E1C2S2	19.68a	2.95a	4.95a	5.42c	0.10f	14.62f	9.17kl	11.44m	3.03b	6.70c	0.73lm	20.52b	0.00m	0.78e
	E1C2S3	2.45mn	0.06j	0.07l	1.76klm	0.10f	3.34kl	39.97a	38.43j	0.15ef	0.27kl	0.65m	12.17i	0.16kl	0.51ij
	E1C2S4	3.05klm	0.23h	0.29ij	2.44gh	3.41a	11.66h	12.37hi	50.92bc	0.11f	2.00j	2.29b	10.66jkl	0.24g	0.34m
	E1C3S1	4.96hi	0.69e	0.77e	4.60de	1.09d	35.70a	10.88ijk	15.44l	2.62c	5.11f	1.82f	15.35f	0.19jk	0.77e
	E1C3S2	3.26jkl	0.09j	0.11kl	2.95f	0.20e	4.59jk	22.92e	53.62ab	0.17ef	0.00l	1.40ij	10.28kl	0.23g	0.37lm
	E1C3S3	5.60h	0.30gh	0.44ghi	1.33no	0.10f	5.80j	18.03f	44.14fgh	0.18ef	2.78h	1.50hi	18.37cde	0.18jk	1.34b
	E1C3S4	15.55c	2.56b	4.34b	5.68c	1.12d	20.25d	10.23jkl	7.31n	3.11b	9.46a	0.79lm	19.28bc	0.00m	0.33m
	E2C1S1	3.29jkl	0.07j	0.09l	2.02ijk	0.10f	3.19l	37.16b	41.08hij	0.15ef	0.27kl	0.84l	11.14ijkl	0.19j	0.51ij
	E2C1S2	3.05klm	0.21h	0.27jk	1.88jkl	0.10f	12.32gh	15.32g	47.28def	0.28e	2.29ij	2.45a	13.60gh	0.35e	0.69fg
	E2C1S3	9.57e	1.32d	1.40d	4.39e	0.02f	27.31b	11.45hij	15.30l	2.37d	6.34d	1.46i	18.07cde	0.28f	0.74ef
	E2C1S4	2.56lmn	0.06j	0.10kl	2.57g	0.10f	3.09l	30.48c	46.99def	0.15ef	0.00l	1.40ij	11.35ijk	0.62a	0.63gh
	E2C2S1	4.81i	0.29gh	0.37hij	1.60lmn	0.10f	4.51jkl	17.50f	51.63bc	0.22ef	2.14j	1.29j	14.63fg	0.16kl	0.86cd
	E2C2S2	18.06b	3.01a	4.82a	6.66a	2.04b	13.52fg	11.41hij	11.22mn	2.35d	8.11b	0.69m	17.32de	0.20ij	0.58hi
	E2C2S3	2.05n	0.04j	0.06l	2.15hij	0.08f	3.18l	39.33a	38.86ij	0.19ef	0.29kl	0.64m	12.43hi	0.15l	0.61h
	E2C2S4	5.38hi	0.38g	0.44ghi	1.16o	0.06f	9.23i	13.09h	50.44bcd	0.14ef	2.63hi	2.13cd	14.05fg	0.23gh	0.69fg
	E2C3S1	3.89j	0.54f	0.76e	4.80d	0.07f	25.67c	13.42h	20.55k	2.47d	5.11f	2.03de	19.39bc	0.45c	0.92c
	E2C3S2	2.67lmn	0.11ij	0.11kl	2.50g	0.10f	3.56kl	23.43e	55.31a	0.12ef	0.00l	1.52hi	9.91l	0.35e	0.42kl
	E2C3S3	6.29g	0.39g	0.59fg	1.05o	0.07f	5.46j	16.67fg	44.78efg	0.15ef	2.56hi	1.74fg	18.69cd	0.22ghi	1.40ab
	E2C3S4	14.25d	2.30c	3.54c	6.24b	1.07d	17.48e	8.35l	11.38m	3.76a	5.94e	0.63m	24.25a	0.00m	0.80de

EC (environmental conditions); Cult (cultivar); SA (salicylic acid); E1: normal; E2: heat stress; C1: Bushehr cultivar; C2: Bona cultivar; C3: Bodegold cultivar; S1: 0 mg·L^−1^ SA; S2: 10 mg·L^−1^ SA; S3: 25 mg·L^−1^ SA; S4: 100 mg·L^−1^ SA; T-b-farn: *trans*-*β*-farnesene; Germ-D: germacerene D; Germ-B: germacerene B; Chama: chamazulene; Nero: nerolidol; Spath: spathulenol; 3-Methyl-A: 3-Methyl-thiophene-2-carboxamide A; *Cis*-dicyclo: *cis*-en-yn-dicycloether; 3-Methyl-B: 3-Methyl-thiophene-2-carboxamide B; *Trans*-dicyclo: *trans*-en-yn-dicycloether. Means followed by the same letters in each column and each row are not significantly different at (*p* ≤ 0.05).

## Supplementary Materials

The following are available online at www.mdpi.com/2304-8158/5/3/56/s1, Table S1: Mean comparison of the simple effects (environmental conditions, cultivar and salicylic acid) and the interaction of environmental conditions × cultivar and environmental conditions × salicylic acid on chemical compounds of German chamomile, Table S2: Mean comparison of the interaction of cultivar × salicylic acid on chemical compounds of German chamomile.
