# Correction: Ghasemi, M., et al. Increase of Chamazulene and α-Bisabolol Contents of the Essential Oil of German Chamomile (*Matricaria chamomilla* L.) Using Salicylic Acid Treatments under Normal and Heat Stress Conditions *Foods* 2016, *5*, 56

**DOI:** 10.3390/foods6020011

**Published:** 2017-02-08

**Authors:** Mojtaba Ghasemi, Nadali Babaeian Jelodar, Mohammad Modarresi, Nadali Bagheri, Abbas Jamali

**Affiliations:** 1 Department of Research and Development, Sea Bioprospecting Co., Ltd., Tangestan Science and Technology Incubator, Persian Gulf Science and Technology Park, Bushehr 7515797414, Bushehr, Iran; 2 Department of Plant Breeding and Biotechnology, Faculty of Crop Science, Sari Agricultural Sciences and Natural Resources University, Sari 578, Mazandaran, Iran; n.babaeian@sanru.ac.ir (N.B.J.); n.bagheri@sanru.ac.ir (N.B.); 3 Department of Plant Breeding, Faculty of Agriculture and Natural Resources, Persian Gulf University, Bushehr 7561158818, Bushehr, Iran; modarresi@pgu.ac.ir; 4 The Persian Gulf Research and Study Institute, Persian Gulf University, Bushehr 7561158818, Bushehr, Iran; a.jamali111@yahoo.com

The authors wish to make the following corrections to their paper [1]. The chamomile scientific name should be *Matricaria chamomilla* instead of *Matricaria chamomila* in the title and main text of this paper. Besides, the authors wish to remove Affiliation 5 (Young Researchers and Elite Club, Bushehr Branch, Islamic Azad University, Bushehr 751961955, Iran). Affiliation 1 has been changed from ‘Tangestan Growth and Technology Center’ to ‘Tangestan Science and Technology Incubator’.

The authors would like to apologize for any inconvenience caused to the readers by these changes. The change does not affect the scientific results. The manuscript will be updated and the original will remain online on the article webpage.

## References

[1] Ghasemi M., Babaeian Jelodar N., Modarresi M., Bagheri N., Jamali A. (2016). Increase of Chamazulene and α-Bisabolol Contents of the Essential Oil of German Chamomile (*Matricaria chamomilla* L.) Using Salicylic Acid Treatments under Normal and Heat Stress Conditions. Foods.

