# Supplementary material for: Increase of Chamazulene and α-Bisabolol Contents of the Essential Oil of German Chamomile (Matricaria chamomila L.) Using Salicylic Acid Treatments under Normal and Heat Stress Conditions
Source: Foods. 2016 Aug 27;5(3):56. doi: 10.3390/foods5030056 (PMC5302395; doi:10.3390/foods5030056)
Supplement: Supplementary file 1 [file foods-05-00056-s001.doc]

Increase of Chamazulene and α-Bisabolol Contents of the Essential Oil of German Chamomile (*Matricaria chamomila* L.) Using Salicylic Acid Treatments under Normal and Heat Stress Conditions

Mojtaba Ghasemi, Nadali Babaeian Jelodar, Mohammad Modarresi, Nadali Bagheri and Abbas Jamali

**Table S1.** Mean comparison of the simple effects (environmental conditions, cultivar and salicylic acid) and the interaction of environmental conditions × cultivar and environmental conditions × salicylic acid on chemical compounds of German chamomile.

| **Treatments** | | **Chemical compounds** | | | | | | | | | | | | | |
| --- | --- | --- | --- | --- | --- | --- | --- | --- | --- | --- | --- | --- | --- | --- | --- |
|  |  | **T-b-farn (%)** | **Germ-D (%)** | **Germ-B (%)** | **Chama (%)** | **α-Bisabolol (%)** | **Bisabolol oxide B (%)** | **Bisabolone oxide (%)** | **Bisabolol oxide A (%)** | **Nero (%)** | **Spath (%)** | **3-Methyl-A (%)** | ***Cis*-dicyclo (%)** | **3-Methyl-B (%)** | ***Trans*-dicyclo (%)** |
| EC | E1 | 3.94b | 0.36b | 0.43b | 2.95b | 0.56a | 14.34a | 21.45a | 36.07a | 0.96b | 2.68b | 1.63a | 13.76b | 0.29a | 0.65b |
|  | E2 | 8.56a | 1.04a | 1.66a | 3.32a | 0.43b | 8.74b | 17.61b | 35.84a | 1.13a | 3.37a | 1.24b | 16.12a | 0.22b | 0.80a |
| Cult | C1 | 2.48c | 0.07c | 0.11c | 2.34b | 0.10b | 3.24c | 31.07a | 47.08a | 0.15b | 0.46c | 1.29c | 10.72c | 0.43a | 0.57c |
|  | C2 | 5.30b | 0.29b | 0.35b | 1.92c | 0.09b | 10.56b | 16.74b | 45.18b | 0.12b | 2.54b | 1.90a | 13.65b | 0.35b | 1.11a |
|  | C3 | 11.82a | 1.75a | 2.80a | 5.47a | 0.79a | 19.75a | 10.53c | 16.86c | 2.87a | 5.69a | 1.34b | 19.42a | 0.18c | 0.76b |
| SA | S1 | 6.53b | 0.70b | 1.09b | 3.24a | 0.32c | 11.18b | 19.45b | 36.38ab | 1.05b | 2.89b | 1.51a | 14.60b | 0.32a | 0.81a |
|  | S2 | 5.81c | 0.65c | 1.00c | 3.13a | 1.00a | 13.56a | 19.07b | 34.98b | 1.06b | 3.27a | 1.41bc | 14.35b | 0.17d | 0.61c |
|  | S3 | 6.89a | 0.83a | 1.17a | 3.19a | 0.41b | 10.66b | 20.55a | 35.59ab | 0.92c | 3.19a | 1.35c | 14.35b | 0.30b | 0.67b |
|  | S4 | 5.76c | 0.63c | 0.92d | 2.98b | 0.24d | 10.76b | 19.05b | 36.89a | 1.14a | 2.76b | 1.45b | 16.45a | 0.23c | 0.81a |
| EC × Cult | E1C1 | 2.48e | 0.05e | 0.08e | 2.06d | 0.10d | 3.27e | 38.02a | 40.63d | 0.17c | 0.32d | 0.80e | 11.36d | 0.24c | 0.52d |
|  | E1C2 | 3.74d | 0.25d | 0.31d | 1.95d | 0.91b | 11.37c | 14.42d | 49.19b | 0.16c | 2.41c | 2.26a | 12.12c | 0.33b | 0.63c |
|  | E1C3 | 5.60c | 0.78b | 0.89b | 4.83b | 0.67c | 28.39a | 11.91e | 18.39e | 2.54b | 5.31b | 1.81b | 17.78b | 0.32b | 0.79b |
|  | E2C1 | 2.83e | 0.09e | 0.11e | 2.59c | 0.13d | 3.59e | 25.84b | 51.49a | 0.14c | 0.11e | 1.46d | 10.82d | 0.40a | 0.53d |
|  | E2C2 | 5.95b | 0.34c | 0.47c | 1.38e | 0.09d | 6.16d | 17.19c | 45.70c | 0.17c | 2.46c | 1.54c | 17.20b | 0.19d | 1.25a |
|  | E2C3 | 16.89a | 2.70a | 4.41a | 6.00a | 1.08a | 16.47b | 9.79f | 10.34f | 3.06a | 7.55a | 0.71f | 20.34a | 0.05e | 0.62c |
| EC × SA | E1S1 | 3.20f | 0.26d | 0.33e | 3.38b | 0.55c | 13.49c | 21.47a | 38.18a | 1.00c | 2.62f | 1.73a | 12.74e | 0.43a | 0.66de |
|  | E1S2 | 3.49ef | 0.33d | 0.38e | 2.93de | 1.53a | 16.90a | 21.08a | 34.93b | 0.96cd | 2.46f | 1.59b | 12.73e | 0.20de | 0.54f |
|  | E1S3 | 5.31d | 0.53c | 0.59d | 2.76ef | 0.07f | 14.27b | 21.31a | 34.56b | 0.93cd | 2.97d | 1.58b | 14.27d | 0.27c | 0.65e |
|  | E1S4 | 3.77e | 0.32d | 0.42e | 2.70f | 0.07f | 12.69d | 21.95a | 36.62ab | 0.94cd | 2.68ef | 1.60b | 15.29c | 0.28c | 0.74c |
|  | E2S1 | 9.87a | 1.14a | 1.85a | 3.10cd | 0.10f | 8.87f | 17.42c | 34.57b | 1.09b | 3.17c | 1.29c | 16.45b | 0.21d | 0.97a |
|  | E2S2 | 8.14b | 0.98b | 1.63b | 3.32b | 0.47d | 10.21e | 17.06cd | 35.02b | 1.16b | 4.08a | 1.23c | 15.98bc | 0.14f | 0.68de |
|  | E2S3 | 8.48b | 1.12a | 1.76a | 3.61a | 0.75b | 7.04g | 19.80b | 36.61ab | 0.91d | 3.42b | 1.13d | 14.44d | 0.33b | 0.69d |
|  | E2S4 | 7.74c | 0.93b | 1.41c | 3.26bc | 0.41e | 8.83f | 16.15d | 37.16a | 1.35a | 2.83de | 1.30c | 17.62a | 0.19e | 0.87b |

EC (environmental conditions), Cult (cultivar), SA (salicylic acid), E1: normal, E2: heat stress, C1: Bushehr cultivar, C2: Bona cultivar, C3: Bodegold cultivar, S1: 0 mg·L^-1^ SA,
S2: 10 mg·L^-1^ SA, S3: 25 mg·L^-1^ SA, S4: 100 mg·L^-1^ SA. T-b-farn: *trans*-β-farnesene, Germ-D: germacerene D, Germ-B: germacerene B, Chama: chamazulene, Nero: nerolidol, Spath: spathulenol, 3-Methyl-A: 3-Methyl-thiophene-2-carboxamide A, *Cis*-dicyclo: *cis*-en-yn-dicycloether, 3-Methyl-B: 3-Methyl-thiophene-2-carboxamide B, *Trans*-dicyclo: *trans*-en-yn-dicycloether. Means followed by the same letters in each column and each row are not significantly different at *p* ≤ 0.05.

**Table S2.** Mean comparison of the interaction of cultivar × salicylic acid on chemical compounds of German chamomile.

| **Treatments** | | **Chemical compounds** | | | | | | | | | | | | | |
| --- | --- | --- | --- | --- | --- | --- | --- | --- | --- | --- | --- | --- | --- | --- | --- |
|  |  | **T-b-farn (%)** | **Germ-D (%)** | **Germ-B (%)** | **Chama (%)** | **α-Bisabolol (%)** | **Bisabolol oxide B (%)** | **Bisabolone oxide (%)** | **Bisabolol oxide A (%)** | **Nero (%)** | **Spath (%)** | **3-Methyl-A (%)** | ***Cis*-dicyclo (%)** | **3-Methyl-B (%)** | ***Trans*-dicyclo (%)** |
| Cult × SA | C1S1 | 2.48hi | 0.07g | 0.11g | 2.34c | 0.10fg | 3.24g | 31.07b | 47.08ab | 0.15de | 0.46e | 1.29b | 10.72f | 0.43a | 0.57f |
|  | C1S2 | 5.30f | 0.29f | 0.35f | 1.92d | 0.09g | 10.56d | 16.74c | 45.18bc | 0.12e | 2.54c | 1.90a | 13.65e | 0.35c | 1.11a |
|  | C1S3 | 11.82b | 1.75b | 2.80b | 5.47a | 0.79d | 19.75c | 10.53f | 16.86d | 2.87b | 5.69b | 1.34b | 19.42b | 0.18h | 0.76d |
|  | C1S4 | 2.86hi | 0.07g | 0.09g | 2.35c | 0.15f | 3.96g | 31.45b | 46.03bc | 0.16de | 0.14f | 1.03c | 11.23f | 0.20g | 0.44h |
|  | C2S1 | 4.33g | 0.27f | 0.37f | 1.89d | 1.75a | 8.73e | 15.20de | 47.53ab | 0.14de | 2.39cd | 1.90a | 14.51e | 0.21fg | 0.84c |
|  | C2S2 | 10.25c | 1.62c | 2.55c | 5.14b | 1.11b | 27.98a | 10.56f | 11.37e | 2.87b | 7.29a | 1.30b | 17.31c | 0.09i | 0.55fg |
|  | C2S3 | 2.92h | 0.06g | 0.10g | 2.30c | 0.10fg | 3.14g | 33.82a | 44.04c | 0.15de | 0.13f | 1.12c | 11.24f | 0.41b | 0.57f |
|  | C2S4 | 3.93g | 0.25f | 0.32f | 1.74d | 0.10fg | 8.42e | 16.41cd | 49.45a | 0.25d | 2.22d | 1.87a | 14.11e | 0.25d | 0.77d |
|  | C3S1 | 13.82a | 2.16a | 3.11a | 5.52a | 1.03c | 20.42c | 11.43f | 13.26e | 2.36c | 7.22a | 1.07c | 17.70c | 0.24de | 0.66e |
|  | C3S2 | 2.36i | 0.07g | 0.08g | 2.32c | 0.09fg | 3.37g | 31.38b | 47.09ab | 0.16de | 0.15f | 1.08c | 11.17f | 0.25d | 0.51g |
|  | C3S3 | 5.84e | 0.38e | 0.52e | 1.11e | 0.07g | 7.34f | 14.88e | 47.61ab | 0.14de | 2.60c | 1.94a | 16.37d | 0.23ef | 1.04b |
|  | C3S4 | 9.07d | 1.42d | 2.15d | 5.52a | 0.57e | 21.57b | 10.88f | 15.96d | 3.12a | 5.53b | 1.33b | 21.82a | 0.22ef | 0.86c |

EC (environmental conditions), Cult (cultivar), SA (salicylic acid), E1: normal, E2: heat stress, C1: Bushehr cultivar, C2: Bona cultivar, C3: Bodegold cultivar, S1: 0 mg·L^-1^ SA,
S2: 10 mg·L^-1^ SA, S3: 25 mg·L^-1^ SA, S4: 100 mg·L^-1^ SA. T-b-farn: *trans*-β-farnesene, Germ-D: germacerene D, Germ-B: germacerene B, Chama: chamazulene, Nero: nerolidol, Spath: spathulenol, 3-Methyl-A: 3-Methyl-thiophene-2-carboxamide A, *Cis*-dicyclo: *Cis*-en-yn-dicycloether, 3-Methyl-B: 3-Methyl-thiophene-2-carboxamide B, *Trans*-dicyclo: *trans*-en-yn-dicycloether. Means followed by the same letters in each column and each row are not significantly different at *p* ≤ 0.05.
